# Supplementary material for: Anti-CTLA4 treatment reduces lymphedema risk potentially through a systemic expansion of the FOXP3+ Treg population
Source: Nat Commun. 2024 Dec 30;15:10784. doi: 10.1038/s41467-024-55002-6 (PMC11686037; doi:10.1038/s41467-024-55002-6)
Supplement: Supplementary file 1 — Supplementary Information [file 41467_2024_55002_MOESM1_ESM.pdf]

Anti-CTLA4 treatment reduces lymphedema risk through a systemic expansion of the FOXP3+ T_{reg} population

Authors: Stefan Wolf[1], Matiar Madanchi[2], Patrick Turko[2,3], Maija Hollmén[4], Sonia Tugues[5], Julia von Atzigen[1], Pietro Giovanoli[1], Reinhard Dummer[2], Nicole Lindenblatt[1], Cornelia Halin[6], Michael Detmar[6], Mitchell Levesque[2], Epameinondas Gousopoulos[1]

Affiliations:

[1]Division of Plastic Surgery, University Hospital Zurich, University of Zurich, 8091 Zurich, Switzerland.
[2]Division of Dermatology, University Hospital Zurich, University of Zurich, 8091 Zurich, Switzerland.
[3]Bioinformatic Department, University Hospital Zurich, University of Zurich, 8091 Zurich, Switzerland.
[4]MediCity Research Laboratory, University of Turku, 20520 Turku, Finland.
[5]Institute of Experimental Immunology, University of Zurich, 8057 Zurich, Switzerland.
[6]Institute of Pharmaceutical Sciences, ETH Zurich, 8093 Zurich, Switzerland.

Inventory of Supplementary Information

Suppl. Figure1: Stage depended evaluation of the immune cell infiltration in lymphedematous tissue
Suppl. Figure 2: Upregulated "Toxic Pathologies" in mice two weeks after lymphedema induction
Suppl. Figure 3: Flow cytometry gating example for single cells isolated lymphedematous tissue and PBMCs from mice and humans
Suppl. Figure 4. Anti-CTLA4 treatment exhibits efficacy in the lymphedema mouse tail model
Suppl. Figure 5: AUC quantification of tail volume in BALB/c and C57BL/6 mice.
Suppl. Figure 6: Absolute cell count measurement of Treg cells in blood and edematous skin tail tissue.
Suppl. Table 1: Localization of lymphedema in 81 melanoma patients
Suppl. Table 2: Lymphedema development and number of lymph nodes removed in melanoma patients undergoing sentinel lymph node biopsy
Suppl. Table 3: Patient characteristics of melanoma patients undergoing lymphadenectomy
Suppl. Table 4: Patient characteristics of each melanoma patient underwent lymphadenectomy and developed lymphedema
Suppl. Table 5: Comparison of treated and untreated melanoma patients which underwent lymphadenectomy and developed lymphedema
Suppl. Table 6: Comparison of melanoma patients, treated with different immunotherapies, which underwent lymphadenectomy and developed lymphedema
Suppl. Table 7: Lymphedema patient characteristics from the patients that have been used for histological and molecular characterization
Suppl. Table 8: Human primer list used for qPCRs
Suppl. Table 9: Murine primer list used for qPCRs
Suppl. Table 10: List of mouse flow cytometry antibodies
Suppl. Table 11: List of human flow cytometry antibodies
Suppl. Table 12: Treatment details of Anti CTLA4 treated melanoma patients
Reference List

Supplementary Material

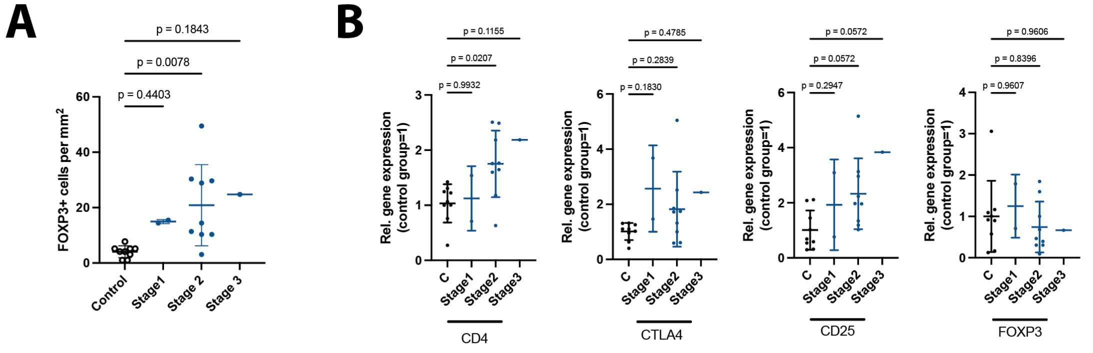

Suppl. Figure 1: Stage depended evaluation of the immune cell infiltration in lymphedematous tissue (A) Evaluation of stage depended FOXP3+ quantification **(B)** Evaluation of stage depended evaluation of CD4, CTLA4, CD25 and FOXP3 mRNA expression in human subcutaneous fat tissue (N(C)=9 patients and N(LE)=12 patients and significance was determined via an one way ANOVA test followed by a Dunnet test was used. Line represent mean ± SD of each group.

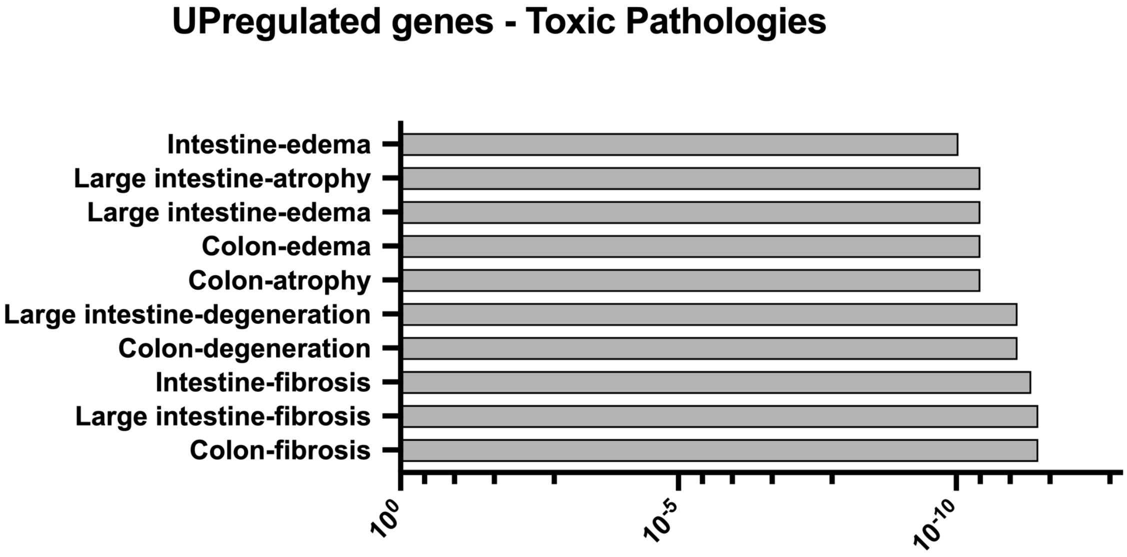

Suppl. Figure 2: Upregulated "Toxic Pathologies" in mice two weeks after lymphedema induction. Re-analysis of the RNA sequencing in C57BL/6 mice two weeks after lymphedema induction, using the data obtained by Gousopoulos *et* al. [1] and the MetaCore "Toxic Pathologies" revealed the key pathways involved in lymphedema development, namely fibrosis, tissue degeneration and edema. These pathways are the major pathways downregulated by anti-CTLA4 treatment in Figure 5H.

These key hallmarks are all related to intestine and colon tissue, because fibrosis and edema in mouse skin and soft tissue are understudied, thus lacking the appropriate number of reference genes. According to MetaCore, colon edema is represented with 1259 reference genes, while skin edema counts 110 references. Similarly, colon fibrosis has 913 gene references, while skin fibrosis 84.

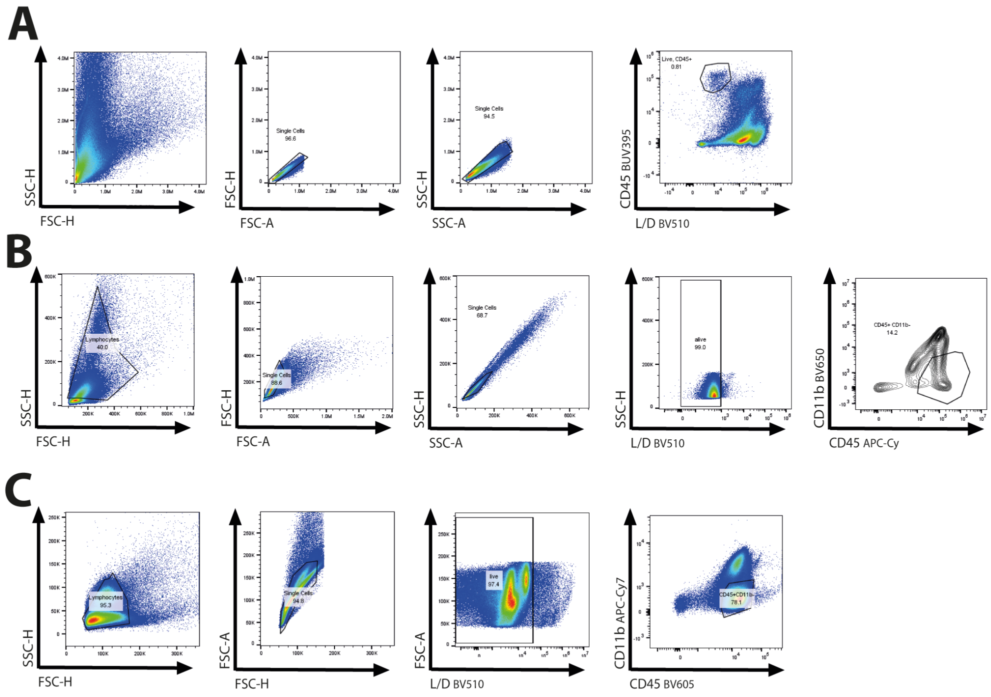

Suppl. Figure 3: Flow cytometry gating example for single cells isolated lymphedematous tissue (Figure 5 A-D) (A) and PBMCs from mice (Figure 4 E-F) (B) and humans (Figure 6 A-D) (C)

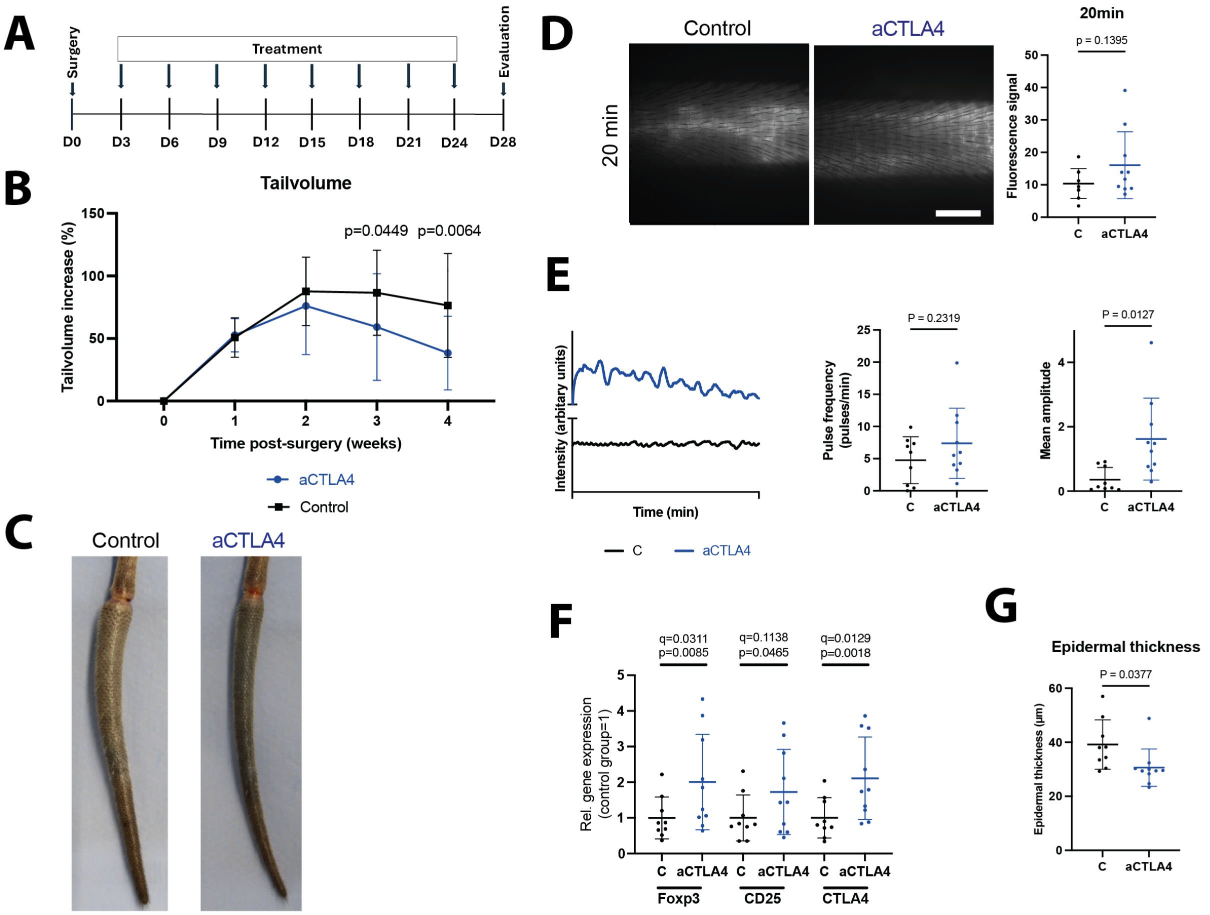

Suppl. Figure 4: **Anti-CTLA4 treatment exhibits efficacy in the lymphedema mouse tail model** (**A**) Surgery was performed on day 0 (D0), treatment was given on day 3, 6, 9, 12, 15, 18 and 24 (D3, D6, D9, D12, D15, D18, D21, D24) and evaluation/endpoint was in day 28 (D28). **(B)** Anti-CTLA4 administration (aCTLA4) following the surgical induction of lymphedema leads to significantly reduced edema 3 and 4 weeks (W) after surgery. **(C)** Representative photographs of tails of control and anti-CTLA4 (aCTLA4)–treated mice 4 weeks postoperatively. **(D)** Representative images of near-infrared intravital microscopy of the tail lymphatic network approx. 1.5 cm distally to the surgical site, visualized through the uptake and transport of a lymphatic-specific fluorescent tracer 20 minutes after infusion near the tip of the tail. Quantification of lymphatic vascular transport based on fluorescence intensity. Scale bar: 2000 µm **(E)** Graphical representation of lymphatic vessel pulsations in the tail collecting vessels of mice treated with or without aCTLA4 4 weeks. Quantification of pulsation frequency and amplitude is shown to the right **(F)** Gen expression analysis from Treg related genes. **(G)** Quantification of the epidermal thickness

All experiments show data from 9 control mice (C) and 10 aCTLA4 treated mice (aCTLA4). Line represent mean ± SD of each group. Significance was determined via a 2-way ANOVA followed by a Bonferroni's multiple comparisons test (B), a Welch's *t*-test (D, E) and an ANOVA and the pairwise multiple comparison analysis was corrected for multiple comparisons with the method of Benjamini, Krieger and Yekutieli (F).

Significance was determined via Bonferroni's multiple comparisons test (B), Welch's *t*-test (D,E and G) and ANOVA and the pairwise multiple comparison analysis was corrected for multiple comparisons the method of Benjamini, Krieger and Yekutieli (D).).

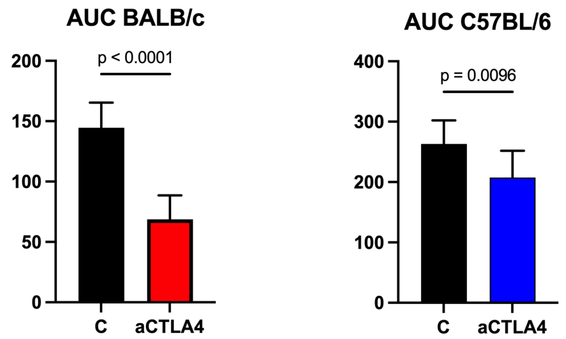

Suppl. Figure 5: AUC quatification of tail volume in BALB/c and C57BL/6 mice. Significance was determined via Welch's *t*-test. Data show mean ± SD of each group.

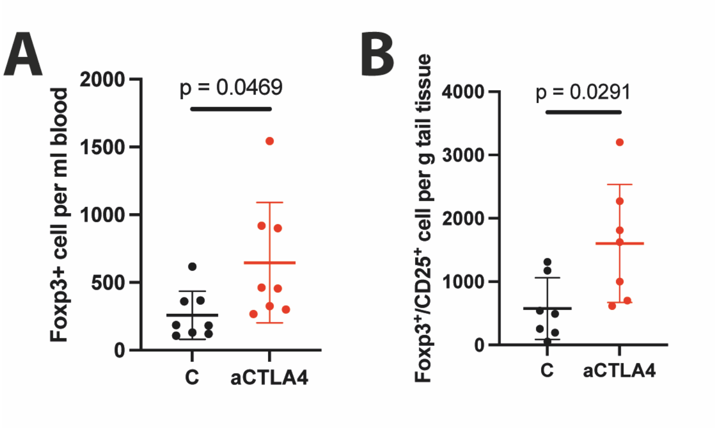

Suppl. Figure 6: Absolute cells count measurement of T_{reg} cells in blood. (N(C)=8 mice and N(aCTLA4)=8 mice (A) and edematous skin tail tissue (N(C)=7 mice and N(aCTLA4)=7 mice). (B). Significance was determined via Welch's *t*-test. Line represent mean ± SD of each group.

Suppl. Table 1: Localization of lymphedema in 81 melanoma patients

Localisation Lymphedema	N° of patients
**Upper extremity**	21(26,0%)
**Lower extremity**	57 (70.4%)
**Upper + Lower extremities**	1 (1.2%)
**Neck**	2 (2.5%)

Suppl. Table 2: Lymphedema development and number of lymph nodes removed in melanoma patients undergoing sentinel lymph node biopsy (SLNB)

LE after SLNB	N° of patients
Total number	13
Anatomic localization of LE:<br>Upper extremity<br>Lower extremity	 1<br>12
N° of lymph nodes (LN) removed:<br>1 LN<br>2 LN<br>3 LN<br>4 LN	 8<br>2<br>2<br>1

Suppl. Table 3: Patient characteristics of melanoma patients undergoing lymphadenectomy (LAD). A multivariate generalized linear model (logistic link) was applied to assess the influence of age, sex, Clark-level, Breslow thickness, localisation, presence of ulceration, primary diagnosis, and immunotherapy, indicating strongly that only immunotherapy is significantly associated with the probability of developing lymphedema.

	NO Lymphedema	Lymphedema	p-value
n	411	68	
Age (mean (SD))	63.30 ± 14.58	62.63 ± 14.20	0.446
Sex = M (%)	252 (61.3 %)	33 (48.5 %)	0.727
Breslow (mean (SD))	3.61 ± 3.26	2.96 ± 2.78	0.265
Clark.Level (mean (SD))	3.85 ± 0.69	3.84 ± 0.69	0.356
Diagnosis (%)			0.597
Cerebral metastatic melanoma	41 (10.0 %)	3 (4.4 %)	
Lymphoid metastasic melanoma	0 (0.0 %)	1 (1.5 %)	
Metastatic melanoma	315 (76.6 %)	52 (76.5 %)	
Primary cutaneous melanoma	55 (13.4 %)	12 (17.6 %)	
Localization (%)			0.486
	21 (5.1 %)	4 (5.9 %)	
arm	77 (18.7 %)	10 (14.7 %)	
foot left	1 (0.2 %)	0 (0.0 %)	
head	38 (9.2 %)	2 (2.9 %)	
leg	123 (29.9 %)	43 (63.2 %)	
trunk	151 (36.7 %)	9 (13.2 %)	
Ulceration (%)	104 (28.8 %)	12 (20.3 %)	0.738
Immunotherapy	238 (89.5%)	28 (10.5%)	0.0005

Suppl. Table 4: Patient characteristics of each melanoma patient underwent lymphadenectomy and developed lymphedema.

Pat. No	Treatment	Month from LAD to diagnosis	Diagnosis performed by	Lymphedema Stage	Diagnosis methode
1	No immunotherapy	1	Angiologist	Penoscrotal edema	Sonography
2	No immunotherapy	2	Dermatologist	Stage 2	Sonography
3	No immunotherapy	2	Plastic surgeon	Stage 2	MRI
4	No immunotherapy	3	Dermatologist	Stage 2	Sonography
5	No immunotherapy	1	Dermatologist	Stage 2	Sonography
6	No immunotherapy	1	Plastic surgeon	Stage 2	CT
7	No immunotherapy	1	Plastic surgeon	Stage 3	MRI
8	No immunotherapy	4	Angiologist	Stage 3	CT
9	No immunotherapy	3	Plastic surgeon	Stage 3	MRI
10	No immunotherapy	8	Plastic surgeon	Stage 3	MRI
11	No immunotherapy	1	Plastic surgeon	Stage 3	CT
12	No immunotherapy	4	Dermatologist	Stage 2	CT
13	No immunotherapy	5	Dermatologist	Stage 3	MRI
14	No immunotherapy	1	Plastic surgeon	Stage 2	Sonography
15	No immunotherapy	2	Plastic surgeon	Stage 3	Sonography
16	No immunotherapy	3	Angiologist	Stage 3	CT
17	No immunotherapy	1	Dermatologist	Stage 3	MRI

18	No immunotherapy	5	Plastic surgeon	Stage 3	CT
19	No immunotherapy	1	Dermatologist	Stage 3	CT
20	No immunotherapy	1	Plastic surgeon	Stage 2	MRI
21	No immunotherapy	7	Dermatologist	Stage 2	CT
22	No immunotherapy	48	Angiologist	Stage 2	Sonography
23	No immunotherapy	2	Plastic surgeon	Stage 2	MRI
24	No immunotherapy	12	Angiologist	Stage 3	Sonography
25	No immunotherapy	1	Angiologist	Stage 2	Sonography
26	No immunotherapy	1	Plastic surgeon	Stage 2	MRI
27	No immunotherapy	2	Plastic surgeon	Stage 3	MRI
28	No immunotherapy	1	Plastic surgeon	Stage 3	MRI
29	No immunotherapy	1	Dermatologist	Stage 3	CT
30	No immunotherapy	1	Plastic surgeon	Stage 2	CT
31	No immunotherapy	1	Angiologist	Stage 3	Sonography
32	No immunotherapy	2	Plastic surgeon	Stage 2	MRI
33	No immunotherapy	1	Plastic surgeon	Stage 3	MRI
34	No immunotherapy	3	Plastic surgeon	Stage 2	CT
35	No immunotherapy	10	Angiologist	Stage 2	Sonography
36	No immunotherapy	3	Dermatologist	Stage 2	CT
37	No immunotherapy	1	Dermatologist	Stage 3	MRI

38	No immunotherapy	1	Angiologist	Stage 3	Sonography
39	No immunotherapy	3	Dermatologist	Stage 3	MRI
40	No immunotherapy	3	Angiologist	Stage 3	Sonography
41	Anti-CTLA4	3	Plastic surgeon	Stage 2	CT
42	Anti-CTLA4	3	Plastic surgeon	Stage 3	MR
43	Anti-PD1	2	Plastic surgeon	Stage 2	MRI
44	Anti-PD1	9	Angiologist	Stage 3	CT
45	Anti-PD1	8	Dermatologist	Stage 3	Sonography
46	Anti-PD1	6	Dermatologist	Stage 3	CT
47	Anti-PD1	7	Dermatologist	Stage 3	MRI
48	Anti-PD1	2	Angiologist	Stage 2	Sonography
49	Anti-PD1	11	Dermatologist	Stage 2	CT
50	Anti-PD1	1	Plastic surgeon	Stage 3	MRI
51	Anti-PD1	1	Plastic surgeon	Stage 2	MRI
52	Anti-PD1	5	Angiologist	Stage 2	Sonography
53	Anti-PD1	2	Plastic surgeon	Stage 2	MRI
54	Anti-PD1 + Anti-CTLA4	6	Plastic surgeon	Stage 3	MRI
55	Anti-PD1 + Anti-CTLA4	1	Dermatologist	Stage 3	Sonography
56	Anti-PD1 + Anti-CTLA4	1	Angiologist	Stage 3	MRI
57	Anti-PD1 + Anti-CTLA4	1	Plastic surgeon	Stage 3	MRI

58	Anti-PD1 + Anti-CTLA4	2	Plastic surgeon	Stage 3	CT
59	Anti-PD1 + Anti-CTLA4	15	Plastic surgeon	Stage 2	CT
60	Anti-PD1 + Anti-CTLA4	8	Dermatologist	Stage 2	CT
61	Anti-PD1 + Anti-CTLA4	31	Dermatologist	Stage 3	MRI
62	Anti-PD1 + Anti-CTLA4	1	Plastic surgeon	Stage 3	CT
63	others	1	Plastic surgeon	Stage 2	Sonography
64	others	4	Dermatologist	edema	CT
65	others	1	Plastic surgeon	Stage 2	MRI
66	others	4	Dermatologist	Stage 3	MRI
67	others	1	Dermatologist	Stage 3	MRI
68	others	2	Dermatologist	Stage 2	MRI

Suppl. Table 5: Comparison of treated and untreated melanoma patients which underwent lymphadenectomy and developed lymphedema. Differences in continuous variables (Time between LAD to diagnosis) were tested with a t-test, while differences in categorical variables were tested with Chi square Test.

	**NO immunotherapy**	**immunotherapy**	**p-value**
**Patient No.**	40	28	
**Time from LAD to diagnosis**	3.85 ± 7.60	4.96 ± 6.24	0.511
**Edema Type**			
Stage 2	18 (45,0%)	13 (46.4 %)	1.00
Stage 3	21 (52.5%)	15 (53.6 %)	
Penoscrotal edema	1 (2.5%)		
**Diagnosis by**			
Plastic surgeon	18(45.0%)	13 (46.4%)	
Angiologist	10 (25.0%)	4 (14.3%)	
Dermatologist	12 (30.0%)	11 (32.3%)	
**Diagnosis Methode**			
CT	12 (30.0%)	9 (32.1%)	
Sonography	13 (32.5%)	5 (17.9%)	
MRI	15 (37.5%)	14 (50%)	

Suppl. Table 6: Comparison of melanoma patients, treated with different immunotherapies, which underwent lymphadenectomy and developed lymphedema. Differences in continuous variables (Time between LAD to diagnosis) were tested with a ANOVA, while differences in categorical variables were tested with Chi square Test.

	NO IT	Anti-CTLA4	Anti-PD1	Anti-PD1 + Anti-CTLA4	Others	p-value
**Patient No.**	40	2	11	9	6	
**Time from LAD to diagnosis**	3.85 ± 7.60	3 ± 0.00	4.91 ± 3.54	7.33 ± 10.06	2.17 ± 1.47	0.651
**Edema Type**						
Stage 2	18 (45,0%)	1 (50.0%)	6 (54.5%)	2 (22.2%)	4 (66.6%)	0.596
Stage 3	21 (52.5%)	1 (50.0%)	5 (45.5%)	7 (77.8%)	2 (33.3%)	
Penoscrotal edema	1 (2.5%)					
**Diagnosis by**						
Plastic surgeon	18(45.0%)	2 (100%)	4 (36.4 %)	5 (55.6%)	2 (33.3%)	
Angiologist	10 (25.0%)		3 (27.3%)	1 (11.1%)		
Dermatologist	12 (30.0%)		4 (36.4 %)	3 (33.3%)	4 (66.6%)	
**Diagnosis Methode**						
CT	12 (30.0%)	1 (50.0%)	3 (27.3%)	4 (44.4%)	1 (16.7%)	
Sonography	13 (32.5%)		3 (27.3%)	1 (11.1%)	1 (16.7%)	
MRI	15 (37.5%)	1 (50.0%)	5 (45.5%)	4 (44.4%)	4 (66.6 %)	

Suppl. Table 7: Lymphedema patient characteristics from the patients that have been used for histological and molecular characterization.

	Control	Lymphedema
Number of cases	9	12
Gender		
Female	7	10
Male	2	2
Average BMI	27.86 ± 4.48	27.12 ± 3.21
Average Age	48.45 ± 9.50	59.21 ± 9.82
Lymphedema Stage		
Stage 1		2
Stage 2		9
Stage 3		1
Harvesting location Lower extremity	8	9
Upper extremity	1	3

Suppl. Table 8: Human primer list used for qPCRs

**Primer**	**Sequence (5' to 3')**
B2M forward	TGT GCT CGC GCT ACT CTC TCT
B2M reverse	CGG ATG GAT GAA ACC CAG ACA
CD4 forward	CCT CCT GCT TTT CAT TGG GCT AGG
CD4 reverse	TGA GGA CAC TGG CAG GTC TTC T
CTLA4 forward	ACG GGA CTC TAC ATC TGC AAG G
CTLA4 reverse	GGA GGA AGT CAG AAT CTG GGC AC
CD25 forward	GAG ACT TCC TGC CTC GTC ACA AC
CD25 reverse	GAT CAG CAG GAA AAC ACA GCC G
FOXP3 forward	CAC TGG TTC ACA CGC ATG TTT
FOXP3 reverse	CAC CCG CAC AAA GCA CTT G

Suppl. Table 9: Murine primer list used for qPCRs

**Primer**	**Sequence (5' to 3')**
Rplp0 forward	AGA TTC GGG ATA TGC TGT TGG C
Rplp0 reverse	TCG GGT CCT AGA CCA GTG TTC
Il2 forward	GCG GCA TGT TCT GGA TTT GAC TC
Il2 reverse	CCA CCA CAG TTG CTG ACT CAT C
Infg forward	CAG CAA CAG CAA GGC GAA AAA GG
Infg reverse	TTT CCG CTT CCT GAG GCT GGA T
Tnfa forward	AGC ACA GAA AGC ATG ATC CG
Tnfa reverse	GTT TGC TAC GAC GTG GGC TA
Il10 forward	ACA GCC GGG AAG ACA ATA ACT
Il10 reverse	GCA GCT CTA GGA GCA TGT GG
Il13 forward	CAC ACA AGA CCA GAC TCC CC
Il13 reverse	CCA GGG ATG GTC TCT CCT CA
Ccr4 forward	TCC TGA CGG ACG TGT ACC T
Ccr4 reverse	CAG ACC TAG TCC AAA AAC CCA C
Ccr8 forward	TTC CTC TAC TTA GGG AGA CAA ATG C
Ccr8 reverse	CAT CCA GGG TGG AAG AAT GG
Ctla4 forward	GTA CCT CTG CAA GGT GGA ACT C
Ctla4 reverse	CCA AAG GAG GAA GTC AGA ATC CG
Foxp3 forward	ACT GGG GTC TTC TCC CTC AA
Foxp3 reverse	CGT GGG AAG GTG CAG AGT AG
CD25 forward	AAG ATG AAG TGT GGG AAA ACG G
CD25 reverse	GGG AAG TCT GTG GTG GTT ATG G

Suppl. Table 10: List of mouse flow cytometry antibodies

Antibody	Clone	Dilution	Cat. Nr.	Company
**Surface Markers**				
CD73-APC-Fire	TY/11.8	1:200	127221	Biolegend
CD45-BUV395	30-F11	1:500	564279	BD
CD11b-BUV661	M1/70	1:500	612977	BD
CD8-BUV805	53-6.7	1:100	612898	BD
LAG3-BV421	C9B7W	1:100	125221	Biolegend
CD44-BV570	IM7	1:200	103037	Biolegend
PD1-BV605	29F.1A12	1:100	135219	Biolegend
CD25-BV650	PC61	1:100	102037	Biolegend
TIM3-BV785	RMT3-23	1:200	119725	Biolegend
NKp46-FITC	29A1.4	1:200	137605	Biolegend
TCRb-PE-Cy5	H57-597	1:300	109209	Biolegend
CD127-PE-Cy7	SB/199	1:200	560733	BD
CD39-PerCP-eFluor 710	24DMS1	1:500	46-0391-80	Thermo
CD19-Spark Blue550	6D5	1:100	115565	Biolegend
CD27-V450	LG.3A10	1:200	561245	BD
CD45-APC-Cy7	30-F11	1:200	103116	Biolegend
CD4-APC	GK1.5	1:200	100412	Biolegend
CD8a-eFluor 450	53-6.7	1:200	48-0081-82	eBioscience
Foxp3- PE	FJK-16s	1:100	12-5773-82	eBioscience
CD11b -BV650	M1/70	1:200	101239	Biolegend
**Intracellular Markers**				
CD4-BUV496	GK1.5	1:100	612952	BD
TCF-Alexa Fluor 647	C63D9	1:200	6709S	CellSignaling
CTLA4-APC-R700	UC10-4F10-11	1:1600	565778	BD
FOXP3-PE-eFlour610	FJK-16s	1:100	61-5773-80	Thermo
TNF-BV711	MP6-XT22	1:100	506349	Biolegend
IFNg-BUV737	XMG1.2	1:100	612769	BD
Ki67-BV480	B56	1:200	566172	BD
TOX-PE	REA473	1:200	130-120-785	Milteny1
FOXP3- PE	FJK-16s	1:100	12-5773-82	eBioscience

Suppl. Table 11: List of human flow cytometry antibodies

Antibody	Clone	Dilution	Cat. Nr.	Company
CD45-BV605	HI30	1:200	304041	Biolegend
CD4-APC	SK3	1:200	344613	Biolegend
CD8a-PerCP/Cy5.5	RPA-T8	1:200	301032	Biolegend
CD25-APC-A700	2A3	1:50	565106	BD
Foxp3- PE/Cy 7	236A/E7	1:100	25-4777-42	Thermo
CD127 – BV650	A019D5	1:400	351325	Biolegend
CD11b -APC/Cyanine7	ICRF44	1:200	301341	Biolegend
CD103-BV421	Ber-ACT8	1:100	563882	BD

Suppl. Table 12: Treatment details of Anti CTLA4 treated melanoma patients

	Sex	Age	No. of Infusions	Duration between Therapy start and PBMC isolation in days	PBMC	Serum
Pat 1	f	67	4	63	x	x
Pat 2	m	35	4	63		x
Pat 3	f	32	4	61	x	
Pat 4	f	50	4	65		x
Pat 5	m	66	4	66		x
Pat 6	f	68	4	68		x
Pat 7	f	72	2	65		x
Pat 8	m	34	4	63	x	
Pat 9	f	47	4	66	x	x
Pat 10	m	49	4	63	x	x
Pat 11	m	39	4	62	x	x
Pat 12	m	50	4	63		x
Pat 13	f	15	4	70		x
Pat 14	f	67	4	63		x
Pat 15	m	62	4	63		x
Pat 16	m	75	4	63		x
Pat 17	f	70	4	64		x
Pat 18	m	46	4	63		x
Pat 19	f	35	4	72	x	

Reference List:

1 Gousopoulos, E. *et al.* Regulatory T cell transfer ameliorates lymphedema and promotes lymphatic vessel function. *JCI Insight* **1**, e89081, doi:10.1172/jci.insight.89081 (2016).
