# Supplementary material for: Anti-CTLA4 treatment reduces lymphedema risk potentially through a systemic expansion of the FOXP3+ Treg population
Source: Nat Commun. 2024 Dec 30;15:10784. doi: 10.1038/s41467-024-55002-6 (PMC11686037; doi:10.1038/s41467-024-55002-6)
Supplement: Supplementary file 2 — Description of Additional Supplementary Files [file 41467_2024_55002_MOESM2_ESM.pdf]

Description of Additional Supplementary Files

Supplementary Movie 1

Control Treatment: Representative imaging of the dynamics of lymphatic tracer arrival and initial lymphatic vessel filling in a region close to the surgical excision margin after 2 weeks. Infusion of 5 μL of P20D800 dye was performed at 0.5 μL/minute for 10 minutes, and videos were acquired for a total of 20 minutes. The tracer shows delayed transport in the collecting lymphatic vessels, followed by limited filling of the lymphatic capillary network.

Supplementary Movie 2

aCTLA4 treatment: Representative imaging of the dynamics of lymphatic tracer arrival and initial lymphatic vessel filling in a region close to the surgical excision margin after 2 weeks. Infusion of 5 μL of P20D800 dye was performed at 0.5 μL/minute for 10 minutes, and videos were acquired for a total of 20 minutes. The tracer shows improved transport in the collecting lymphatic vessels, followed by an improved filling of the lymphatic capillary network.
