# Supplementary material for: Anti-CTLA4 treatment reduces lymphedema risk potentially through a systemic expansion of the FOXP3+ Treg population
Source: Nat Commun. 2024 Dec 30;15:10784. doi: 10.1038/s41467-024-55002-6 (PMC11686037; doi:10.1038/s41467-024-55002-6)
Supplement: Supplementary file 5 — Reporting Summary [file 41467_2024_55002_MOESM5_ESM.pdf]

nature portfolio

Corresponding author(s): Dr. Epameinondas Gousopoulos
epameinondas.gousopoulos@usz.ch

Last updated by author(s): Nov 12, 2024

Reporting Summary

Nature Portfolio wishes to improve the reproducibility of the work that we publish. This form provides structure for consistency and transparency in reporting. For further information on Nature Portfolio policies, see our Editorial Policies and the Editorial Policy Checklist.

Statistics

For all statistical analyses, confirm that the following items are present in the figure legend, table legend, main text, or Methods section.

n/a	Confirmed	
☐	☒	The exact sample size (*n*) for each experimental group/condition, given as a discrete number and unit of measurement
☐	☒	A statement on whether measurements were taken from distinct samples or whether the same sample was measured repeatedly
☐	☒	The statistical test(s) used AND whether they are one- or two-sided<br>*Only common tests should be described solely by name; describe more complex techniques in the Methods section.*
☐	☒	A description of all covariates tested
☐	☒	A description of any assumptions or corrections, such as tests of normality and adjustment for multiple comparisons
☐	☒	A full description of the statistical parameters including central tendency (e.g. means) or other basic estimates (e.g. regression coefficient) AND variation (e.g. standard deviation) or associated estimates of uncertainty (e.g. confidence intervals)
☐	☒	For null hypothesis testing, the test statistic (e.g. *F*, *t*, *r*) with confidence intervals, effect sizes, degrees of freedom and *P* value noted<br>*Give P values as exact values whenever suitable.*
☒	☐	For Bayesian analysis, information on the choice of priors and Markov chain Monte Carlo settings
☒	☐	For hierarchical and complex designs, identification of the appropriate level for tests and full reporting of outcomes
☒	☐	Estimates of effect sizes (e.g. Cohen's *d*, Pearson's *r*), indicating how they were calculated

Our web collection on statistics for biologists contains articles on many of the points above.

Software and code

Policy information about availability of computer code

Data collection	Lymphatic vessel transport was assessed by an Axio Zoom V16 stereomicroscope (Zeiss) equipped with a Prime BSI Express sCMOS Camera (Teledyne Photometrics) and a pE-4000 LED light source (CoolLED) using ZEN 3.3 software. Images were acquired with a Zeiss Axio Scan Z1 equipped with a Hitachi HV-F202FCL camera and ZEN3.3 software and Immunofluorescence stained sections were taken with a Leica DM 5500 Microscope using LAS X software. For the acquisition of human and murine PBMCS a Cytoflex S (Beckman Coulter) apparatus with Cytexpert software (version 2.1.3.22) was used. Single cell suspension from lymphedematous tiussue wereacquired with a Cytek Aurora flow cytometer with SpectroFlo® software (Cytek Biosciences). RNA Sequencing was performed on a Illumina NovaSeq 6000 System.
Data analysis	GraphPad Prism (Version 9.0) was used for creation of graphs and statistic analysis. Statistical analysis of the retrospective study was performed with R version 4.2.0. Pictures were analyzed using Image J 1.53t software. Flow cytometry data were analyzed with FlowJo 10.8.1. For RNA Sequencing quantification of gene level expression done using Kallisto (Version 0.46.1). For the detection of differentially expressed genes, a count based negative binomial model implemented in the software package DESeq2 (R version: 4.1.2, DESeq2 version: 1.34.0) was applied.

For manuscripts utilizing custom algorithms or software that are central to the research but not yet described in published literature, software must be made available to editors and reviewers. We strongly encourage code deposition in a community repository (e.g. GitHub). See the Nature Portfolio guidelines for submitting code & software for further information.

Data

Policy information about availability of data

All manuscripts must include a data availability statement. This statement should provide the following information, where applicable:

- Accession codes, unique identifiers, or web links for publicly available datasets
- A description of any restrictions on data availability
- For clinical datasets or third party data, please ensure that the statement adheres to our policy

All data associated with this study are present in the paper, the Supplementary Materials or the raw data file. RNA-seq data are available in ENA repository, accession number PRJEB64781.

Research involving human participants, their data, or biological material

Policy information about studies with human participants or human data. See also policy information about sex, gender (identity/presentation), and sexual orientation and race, ethnicity and racism.

Reporting on sex and gender	Both, male and female sex patients have been included in the analysis (retrospective analysis of melanoma patient registry).
Reporting on race, ethnicity, or other socially relevant groupings	The manuscript did not report on race, ethnicity, or other social grouping.
Population characteristics	Patient characteristics (age, sex), characteristics of melanoma, such as type (primary cutaneous melanoma, metastatic melanoma and no melanoma patients), the primary location of the melanoma, the Breslow index, Clark level including the presence/absence of ulceration, if a sentinel lymph node (SLNB) biopsy was performed or not, if a lymphadenectomy (LA) was performed as well as the time period between the two procedures (SLNB and LA). Then we examined based on the detailed registered information whether the patients developed a lymphedema after SLNB or LA and the specialist who diagnosed the disease. At the end we examined if the patient was under immunotherapy or not, and if yes on which scheme.
Recruitment	No active recruitment occurred, as the data was collected based on a retrospective analysis.<br>We reviewed patients' medical records regarding demographics, melanoma history and characteristics including the performance of lymph node biopsy or lymphadenectomy, the diagnosis of lymphedema and presence of an immune checkpoint inhibitor treatment.<br>Special attention has been paid to the accurate identification of lymphedema diagnosis. Given the difficulty in making the correct diagnosis of lymphedema and the risk of incorrect use of this diagnosis, we only considered patients who have received a diagnosis from plastic surgeons, angiologists and dermatologists observing the time passed from the lymphadenectomy to the development of lymphedema. All these patients with diagnosed lymphedema were found to be in regularly under physiotherapy treatment.
Ethics oversight	The retrospective analysis of the melanoma patient cohort as well as the PBMCs collection/ isolation of the same patients was approved by the Cantonal Ethics Committee of the Canton Zürich, Switzerland (IRB approval number: KEK-ZH-Nr. 2014-0193 by the Cantonal Ethics Committee of the Canton Zurich, Switzerland).<br>The tissue collection from lymphedema and control patients was approved by the Cantonal Ethics Committee of the Canton Zurich, Switzerland (KEK-ZH-Nr: 2021-02358 by the Cantonal Ethics Committee of the Canton Zürich, Switzerland).

Note that full information on the approval of the study protocol must also be provided in the manuscript.

Field-specific reporting

Please select the one below that is the best fit for your research. If you are not sure, read the appropriate sections before making your selection.

☒ Life sciences ☐ Behavioural & social sciences ☐ Ecological, evolutionary & environmental sciences

For a reference copy of the document with all sections, see nature.com/documents/nr-reporting-summary-flat.pdf

Life sciences study design

All studies must disclose on these points even when the disclosure is negative.

Sample size	As this represents an exploratory analysis it was not possible to apply power analysis to calculate sample size, but we apply Fermi's method (feasibility and constraints).
Data exclusions	For the animal Study, exclusion criteria were preestablished and mice were excluded from analysis if any of the following incidents occurred upon surgery: (1) self-inflicted mutilation or severe abrasion on the skin, (2) severe infection and (3) loss of blood supply in the tail. Furthermore, Grubb's test was used for the identification and exclusion of outliers, which is displayed in the raw data file.
Replication	All the non-human data of this study were confirmed twice.

Randomization	The mice were randomly allocated to a certain group prior to the operation and when grouped the animals after the single-housing period, to avoid the effect of the cage
Blinding	Image quantification was performed in a blinded fashion.

Behavioural & social sciences study design

All studies must disclose on these points even when the disclosure is negative.

Study description	*Briefly describe the study type including whether data are quantitative, qualitative, or mixed-methods (e.g. qualitative cross-sectional, quantitative experimental, mixed-methods case study).*
Research sample	*State the research sample (e.g. Harvard university undergraduates, villagers in rural India) and provide relevant demographic information (e.g. age, sex) and indicate whether the sample is representative. Provide a rationale for the study sample chosen. For studies involving existing datasets, please describe the dataset and source.*
Sampling strategy	*Describe the sampling procedure (e.g. random, snowball, stratified, convenience). Describe the statistical methods that were used to predetermine sample size OR if no sample-size calculation was performed, describe how sample sizes were chosen and provide a rationale for why these sample sizes are sufficient. For qualitative data, please indicate whether data saturation was considered, and what criteria were used to decide that no further sampling was needed.*
Data collection	*Provide details about the data collection procedure, including the instruments or devices used to record the data (e.g. pen and paper, computer, eye tracker, video or audio equipment) whether anyone was present besides the participant(s) and the researcher, and whether the researcher was blind to experimental condition and/or the study hypothesis during data collection.*
Timing	*Indicate the start and stop dates of data collection. If there is a gap between collection periods, state the dates for each sample cohort.*
Data exclusions	*If no data were excluded from the analyses, state so OR if data were excluded, provide the exact number of exclusions and the rationale behind them, indicating whether exclusion criteria were pre-established.*
Non-participation	*State how many participants dropped out/declined participation and the reason(s) given OR provide response rate OR state that no participants dropped out/declined participation.*
Randomization	*If participants were not allocated into experimental groups, state so OR describe how participants were allocated to groups, and if allocation was not random, describe how covariates were controlled.*

Ecological, evolutionary & environmental sciences study design

All studies must disclose on these points even when the disclosure is negative.

Study description	*Briefly describe the study. For quantitative data include treatment factors and interactions, design structure (e.g. factorial, nested, hierarchical), nature and number of experimental units and replicates.*
Research sample	*Describe the research sample (e.g. a group of tagged Passer domesticus, all Stenocereus thurberi within Organ Pipe Cactus National Monument), and provide a rationale for the sample choice. When relevant, describe the organism taxa, source, sex, age range and any manipulations. State what population the sample is meant to represent when applicable. For studies involving existing datasets, describe the data and its source.*
Sampling strategy	*Note the sampling procedure. Describe the statistical methods that were used to predetermine sample size OR if no sample-size calculation was performed, describe how sample sizes were chosen and provide a rationale for why these sample sizes are sufficient.*
Data collection	*Describe the data collection procedure, including who recorded the data and how.*
Timing and spatial scale	*Indicate the start and stop dates of data collection, noting the frequency and periodicity of sampling and providing a rationale for these choices. If there is a gap between collection periods, state the dates for each sample cohort. Specify the spatial scale from which the data are taken*
Data exclusions	*If no data were excluded from the analyses, state so OR if data were excluded, describe the exclusions and the rationale behind them, indicating whether exclusion criteria were pre-established.*
Reproducibility	*Describe the measures taken to verify the reproducibility of experimental findings. For each experiment, note whether any attempts to repeat the experiment failed OR state that all attempts to repeat the experiment were successful.*
Randomization	*Describe how samples/organisms/participants were allocated into groups. If allocation was not random, describe how covariates were controlled. If this is not relevant to your study, explain why.*
Blinding	*Describe the extent of blinding used during data acquisition and analysis. If blinding was not possible, describe why OR explain why blinding was not relevant to your study.*

Did the study involve field work? ☐ Yes ☒ No

Reporting for specific materials, systems and methods

We require information from authors about some types of materials, experimental systems and methods used in many studies. Here, indicate whether each material, system or method listed is relevant to your study. If you are not sure if a list item applies to your research, read the appropriate section before selecting a response.

Materials & experimental systems

n/a	Involved in the study
☐	☒ Antibodies
☒	☐ Eukaryotic cell lines
☒	☐ Palaeontology and archaeology
☐	☒ Animals and other organisms
☐	☒ Clinical data
☒	☐ Dual use research of concern
☒	☐ Plants

Methods

n/a	Involved in the study
☒	☐ ChIP-seq
☐	☒ Flow cytometry
☒	☐ MRI-based neuroimaging

Antibodies

Antibodies used

The following antibodies were used for treatment, flow cytometry- and histology stainings:
1. Syrian hamster monoclonal anti-mouse CTLA-4 (CD152)(BP0131,Bioxcell)
2. polyclonal Syrian hamster IgG (BP0087,BioXCell)
3. Rat monoclonal APC/Fire 750 anti-mouse CD73 Antibody (127221, Biolegend)
4. Rat monoclonal BUV395 Anti-Mouse CD45 anitbody (564279, BD)
5. Rat monoclonal BUV661 Anti-CD11b Antibody (612977 BD)
6. Rat monoclonal BUV805 Anti-Mouse CD8a (612898, BD)
7. Rat monoclonal Brilliant Violet 421™ anti-mouse CD223 (LAG-3) Antibody (125221, Biolegend)
8. Rat monoclonal Brilliant Violet 570™ anti-mouse/human CD44 Antibody (103037 Biolegend)
9. Rat monoclonal Brilliant Violet 605™ anti-mouse CD279 (PD-1) Antibody (135219 Biolegend)
10. Rat monoclonal Brilliant Violet 650™ anti-mouse CD25 Antibody (102037 Biolegend)
11. Rat monoclonal Brilliant Violet 785™ anti-mouse CD366 (Tim-3) Antibody (119725 Biolegend)
12. Rat monoclonal FITC anti-mouse CD335 (NKp46) Antibody (137605 Biolegend)
13. Armenian Hamster monoclonal PE/Cyanine5 anti-mouse TCR β chain Antibody (109209 Biolegend)
14. Rat monoclonal PE-Cy7 Anti-Mouse CD127 Antibody (560733 BD)
15. Rat monoclonal PerCP-eFluor 710 CD39 antibody (46-0391-80 Thermo)
16. Rat monoclonal Spark Blue™ 550 anti-mouse CD19 Antibody (115565 Biolegend)
17. Armenian Hamster monoclonal V450 Hamster Anti-Mouse CD27 (561245 BD)
18. Rat monclonal BUV496 Anti-Mouse CD4 (612952 BD)
19. Rabbit monoclonal Alexa Fluor 647 anti-mouse TCF (6709S CellSignaling)
20. Hamster APC-R700 anti-Mouse CD152 Antibody (565778 BD)
21. Rat monoclonal PE-eFlour610 anti-FOXP3 Antibody (61-5773-80 Thermo)
22. Rat monoclonal Brilliant Violet 711™ anti-mouse TNF-α Antibody (506349 Biolegend)
23. Rat monoclonal BUV737 Anti-Mouse IFN-γ Antibody (612769 BD)
24. Mouse monoclonal BV480 Mouse Anti-Ki-67 Anitbody (566172 BD)
25. Human monoclonal PE anti-mouse TOX antibody (130-120-785 Miltenyi)
26. Rat monoclonal APC/Cyanine7 anti-mouse CD45 Antibody (103116 Biolegend)
27. Rat monoclonal APC anti-mouse CD4 Antibody (100412 Biolegend)
28. Rat monoclonal eFluor™ 450 anti mouse CD8a Monoclonal Antibody (48-0081-82 eBioscience
29. Rat monoclonal PE anti-mouse Foxp3 Antibody (12-5773-82 eBioscience)
30. Rat monoclonal Brilliant Violet 650™ anti-mouse/human CD11b Antibody (101239 Biolegend)
31. Mouse monoclonal BV650 Mouse Anti-Human CD45 Antibody (563717 BD)
32. Mouse monoclonal APC anti-human CD4 Antibody (344613, Biolegend)
33. Mouse monoclonal PerCP/Cyanine5.5 anti-human CD8a Antibody (301032 Biolegend)
34. Mouse monoclonal FITC Anti-Human CD25 Antibody (555431 BD)
35. Mouse monoclonal PE/Cy 7 anti-human Foxp3 Antibody (25-4777-42, Thermo)
36. Mouse monoclonal BV421 Anti-Human CD152 Antibody (562743 BD)
37. Mouse monoclonal Brilliant Violet 510™ anti-human CD11b Antibody (301333Biolegend)
38. Rat monoclonal anti-mouse CD16/32 Antibody (101319, Biolegend)
39. Mouse monoclonal anti-human CD4 (IR649, Agilent Dako)
40. Rabbit monoclonal anti-human FOXP3 (abcam, ab215206)
41. Rabbit monoclonal anti-human CTLA4 (abcam, ab237712)
42. Mouse monoclonal Brilliant Violet 605™ anti-human CD45 Antibody (304041 Biolegend)

43. Mouse monoclonal APC-R700 Mouse Anti-Human CD25 (565106 BD)
44. Mouse monoclonal APC/Cyanine7 anti-human CD11b Antibody (301341 Biolegend)
45. Mouse monoclonal Brilliant Violet 650™ anti-human CD127 (IL-7Rα) Antibody (351325 Biolegend)
46, Mouse monoclonal BV421 Mouse Anti-Human CD103(563882 BD)
47. Rabbit monoclonal Anti-human IL2RA (ab231441 Abcam)

Validation

1. Syrian hamster monoclonal anti-mouse CTLA-4 (CD152)(BP0131,Bioxcell)
https://bioxcell.com/invivoplus-anti-mouse-ctla-4-cd152-bp0131
2. polyclonal Syrian hamster IgG (BP0087,BioXCell)
https://bioxcell.com/invivoplus-polyclonal-syrian-hamster-igg-bp0087
3. Rat monoclonal APC/Fire 750 anti-mouse CD73 Antibody (127221, Biolegend)
https://www.biolegend.com/en-us/products/apc-fire-750-anti-mouse-cd73-antibody-14894
4. Rat monoclonal BUV395 Anti-Mouse CD45 anitbody (564279, BD)
https://www.bdbiosciences.com/en-us/products/reagents/flow-cytometry-reagents/research-reagents/single-color-antibodies-ruo/buv395-rat-anti-mouse-cd45.564279
5. Rat monoclonal BUV661 Anti-CD11b Antibody (612977 BD)
https://www.bdbiosciences.com/en-us/products/reagents/flow-cytometry-reagents/research-reagents/single-color-antibodies-ruo/buv661-rat-anti-cd11b.612977
6. Rat monoclonal BUV805 Anti-Mouse CD8a (612898, BD)
https://www.bdbiosciences.com/en-us/products/reagents/flow-cytometry-reagents/research-reagents/single-color-antibodies-ruo/buv805-rat-anti-mouse-cd8a.612898
7. Rat monoclonal Brilliant Violet 421™ anti-mouse CD223 (LAG-3) Antibody (125221, Biolegend)
https://www.biolegend.com/en-us/products/brilliant-violet-421-anti-mouse-cd223-lag-3-antibody-13008
8. Rat monoclonal Brilliant Violet 570™ anti-mouse/human CD44 Antibody (103037 Biolegend)
https://www.biolegend.com/en-us/products/brilliant-violet-570-anti-mouse-human-cd44-antibody-7386
9. Rat monoclonal Brilliant Violet 605™ anti-mouse CD279 (PD-1) Antibody (135219 Biolegend)
https://www.biolegend.com/en-us/products/brilliant-violet-605-anti-mouse-cd279-pd-1-antibody-7648
10. Rat monoclonal Brilliant Violet 650™ anti-mouse CD25 Antibody (102037 Biolegend)
https://www.biolegend.com/en-us/products/brilliant-violet-650-anti-mouse-cd25-antibody-7640
11. Rat monoclonal Brilliant Violet 785™ anti-mouse CD366 (Tim-3) Antibody (119725 Biolegend)
https://www.biolegend.com/en-us/products/brilliant-violet-785-anti-mouse-cd366-tim-3-antibody-14928
12. Rat monoclonal FITC anti-mouse CD335 (NKp46) Antibody (137605 Biolegend)
https://www.biolegend.com/en-us/products/fitc-anti-mouse-cd335-nkp46-antibody-6618
13. Armenian Hamster monoclonal PE/Cyanine5 anti-mouse TCR β chain Antibody (109209 Biolegend)
https://www.biolegend.com/en-us/products/pe-cyanine5-anti-mouse-tcr-beta-chain-antibody-273
14. Rat monoclonal PE-Cy7 Anti-Mouse CD127 Antibody (560733 BD)
https://www.bdbiosciences.com/en-us/products/reagents/flow-cytometry-reagents/research-reagents/single-color-antibodies-ruo/pe-cy-7-rat-anti-mouse-cd127.560733
15. Rat monoclonal PerCP-eFluor 710 CD39 antibody (46-0391-80 Thermo)
https://www.thermofisher.com/antibody/product/CD39-Antibody-clone-24DMS1-Monoclonal/46-0391-80
16. Rat monoclonal Spark Blue™ 550 anti-mouse CD19 Antibody (115565 Biolegend)
https://www.biolegend.com/en-us/products/spark-blue-550-anti-mouse-cd19-antibody-18492
17. Armenian Hamster monoclonal V450 Hamster Anti-Mouse CD27 (561245 BD)
https://www.bdbiosciences.com/content/dam/bdb/products/global/reagents/flow-cytometry-reagents/research-reagents/single-color-antibodies-ruo/561245_base/pdf/561245.pdf
18. Rat monclonal BUV496 Anti-Mouse CD4 (612952 BD)
https://www.bdbiosciences.com/en-us/products/reagents/flow-cytometry-reagents/research-reagents/single-color-antibodies-ruo/buv496-rat-anti-mouse-cd4.612952
19. Rabbit monoclonal Alexa Fluor 647 anti-mouse TCF (6709S CellSignaling)
https://www.cellsignal.com/products/antibody-conjugates/tcf1-tcf7-c63d9-rabbit-mab-alexa-fluor-647-conjugate/6709
20. Hamster APC-R700 anti-Mouse CD152 Antibody (565778 BD)
https://www.bdbiosciences.com/en-us/products/reagents/flow-cytometry-reagents/research-reagents/single-color-antibodies-ruo/apc-r700-hamster-anti-mouse-cd152.565778
21. Rat monoclonal PE-eFlour610 anti-FOXP3 Antibody (61-5773-80 Thermo)
https://www.thermofisher.com/antibody/product/FOXP3-Antibody-clone-FJK-16s-Monoclonal/61-5773-80
22. Rat monoclonal Brilliant Violet 711™ anti-mouse TNF-α Antibody (506349 Biolegend)
https://www.biolegend.com/en-us/products/brilliant-violet-711-anti-mouse-tnf-alpha-antibody-13622
23. Rat monoclonal BUV737 Anti-Mouse IFN-γ Antibody (612769 BD)
https://www.bdbiosciences.com/en-us/products/reagents/flow-cytometry-reagents/research-reagents/single-color-antibodies-ruo/buv737-rat-anti-mouse-ifn.612769
24. Mouse monoclonal BV480 Mouse Anti-Ki-67 Anitbody (566172 BD)
https://www.bdbiosciences.com/en-us/products/reagents/flow-cytometry-reagents/research-reagents/single-color-antibodies-ruo/bv480-mouse-anti-ki-67.566172
25. Human monoclonal PE anti-mouse TOX antibody (130-120-785 Miltenyi)
https://www.miltenyibiotec.com/AT-en/products/tox-antibody-anti-human-mouse-reafinity-rea473.html#conjugate=pe:size=100-tests-in-200-ul
26. Rat monoclonal APC/Cyanine7 anti-mouse CD45 Antibody (103116 Biolegend)
https://www.biolegend.com/en-us/products/apc-cyanine7-anti-mouse-cd45-antibody-2530

27. Rat monoclonal APC anti-mouse CD4 Antibody (100412 Biolegend)
https://www.biolegend.com/en-us/products/apc-anti-mouse-cd4-antibody-245
28. Rat monoclonal eFluor™ 450 anti mouse CD8a Monoclonal Antibody (48-0081-82 eBioscience
https://www.thermofisher.com/antibody/product/CD8a-Antibody-clone-53-6-7-Monoclonal/48-0081-82
29. Rat monoclonal PE anti-mouse Foxp3 Antibody (12-5773-82 eBioscience)
https://www.thermofisher.com/antibody/product/FOXP3-Antibody-clone-FJK-16s-Monoclonal/12-5773-82
30. Rat monoclonal Brilliant Violet 650™ anti-mouse/human CD11b Antibody (101239 Biolegend)
https://www.biolegend.com/en-us/products/brilliant-violet-650-anti-mouse-human-cd11b-antibody-7638
31. Mouse monoclonal BV650 Mouse Anti-Human CD45 Antibody (563717 BD)
https://www.bdbiosciences.com/en-us/products/reagents/flow-cytometry-reagents/research-reagents/single-color-antibodies-ruo/bv650-mouse-anti-human-cd45.563717
32. Mouse monoclonal APC anti-human CD4 Antibody (344613, Biolegend)
https://www.biolegend.com/en-us/products/apc-anti-human-cd4-antibody-6378
33. Mouse monoclonal PerCP/Cyanine5.5 anti-human CD8a Antibody (301032 Biolegend)
https://www.biolegend.com/en-us/products/percp-cyanine5-5-anti-human-cd8a-antibody-4222
34. Mouse monoclonal FITC Anti-Human CD25 Antibody (555431 BD)
https://www.bdbiosciences.com/en-eu/products/reagents/flow-cytometry-reagents/research-reagents/single-color-antibodies-ruo/fitc-mouse-anti-human-cd25.555431
35. Mouse monoclonal PE/Cy 7 anti-human Foxp3 Antibody (25-4777-42, Thermo)
https://www.thermofisher.com/antibody/product/FOXP3-Antibody-clone-236A-E7-Monoclonal/25-4777-42
36. Mouse monoclonal BV421 Anti-Human CD152 Antibody (562743 BD)
https://www.bdbiosciences.com/en-us/products/reagents/flow-cytometry-reagents/research-reagents/single-color-antibodies-ruo/bv421-mouse-anti-human-cd152.562743
37. Mouse monoclonal Brilliant Violet 510™ anti-human CD11b Antibody (301333Biolegend)
https://www.biolegend.com/en-us/products/brilliant-violet-510-anti-human-cd11b-antibody-8544
38. Rat monoclonal anti-mouse CD16/32 Antibody (101319, Biolegend)
https://www.biolegend.com/en-us/antibodies-and-more/trustain-fcx-anti-mouse-cd16-32-antibody-5683?GroupID=BLG9237
39. mouse monoclonal anti-human CD4 (IR649, Agilent Dako)
https://www.agilent.com/en/product/immunohistochemistry/antibodies-controls/primary-antibodies/cd4-(autostainer-link-48)-76373
40. rabbit monoclonal anti-human FOXP3 (abcam, ab215206)
https://www.abcam.com/products/primary-antibodies/foxp3-antibody-epr22102-37-ab215206.html
41. monoclonal rabbit anti-human CTLA4 (abcam, ab237712)
https://www.abcam.com/products/primary-antibodies/ctla4-antibody-cal49-ab237712.html
42. Mouse monoclonal Brilliant Violet 605™ anti-human CD45 Antibody (304041 Biolegend)
https://www.biolegend.com/en-us/products/brilliant-violet-605-anti-human-cd45-antibody-8521
43. Mouse monoclonal APC-R700 Mouse Anti-Human CD25 (565106 BD)
https://www.bdbiosciences.com/en-us/products/reagents/flow-cytometry-reagents/research-reagents/single-color-antibodies-ruo/apc-r700-mouse-anti-human-cd25.565106
44. Mouse monoclonal APC/Cyanine7 anti-human CD11b Antibody (301341 Biolegend)
https://www.biolegend.com/en-us/products/apc-cyanine7-anti-human-cd11b-antibody-9611
45. Mouse monoclonal Brilliant Violet 650™ anti-human CD127 (IL-7Rα) Antibody (351325 Biolegend)
https://www.biolegend.com/en-us/products/brilliant-violet-650-anti-human-cd127-il-7ralpha-antibody-7673
46, Mouse monoclonal BV421 Mouse Anti-Human CD103(563882 BD)
https://www.bdbiosciences.com/en-us/products/reagents/flow-cytometry-reagents/research-reagents/single-color-antibodies-ruo/bv421-mouse-anti-human-cd103.563882
47. Rabbit monoclonal Anti-human IL2RA (ab231441 Abcam)
https://www.abcam.com/en-us/products/primary-antibodies/il-2-receptor-alpha-antibody-sp176-ab231441

Eukaryotic cell lines

Policy information about cell lines and Sex and Gender in Research

Cell line source(s)

State the source of each cell line used and the sex of all primary cell lines and cells derived from human participants or vertebrate models.

Authentication

Describe the authentication procedures for each cell line used OR declare that none of the cell lines used were authenticated.

Mycoplasma contamination

Confirm that all cell lines tested negative for mycoplasma contamination OR describe the results of the testing for mycoplasma contamination OR declare that the cell lines were not tested for mycoplasma contamination.

Commonly misidentified lines
(See ICLAC register)

Name any commonly misidentified cell lines used in the study and provide a rationale for their use.

Palaeontology and Archaeology

Specimen provenance	*Provide provenance information for specimens and describe permits that were obtained for the work (including the name of the issuing authority, the date of issue, and any identifying information). Permits should encompass collection and, where applicable, export.*
Specimen deposition	*Indicate where the specimens have been deposited to permit free access by other researchers.*
Dating methods	*If new dates are provided, describe how they were obtained (e.g. collection, storage, sample pretreatment and measurement), where they were obtained (i.e. lab name), the calibration program and the protocol for quality assurance OR state that no new dates are provided.*

☐ Tick this box to confirm that the raw and calibrated dates are available in the paper or in Supplementary Information.

Ethics oversight	*Identify the organization(s) that approved or provided guidance on the study protocol, OR state that no ethical approval or guidance was required and explain why not.*

Note that full information on the approval of the study protocol must also be provided in the manuscript.

Animals and other research organisms

Policy information about studies involving animals; ARRIVE guidelines recommended for reporting animal research, and Sex and Gender in Research

Laboratory animals	In this study 8- to 12-week-old BALB/c and C57BL/6 female mice ordered from Janvier Labs were used.
Wild animals	The study did not include wild animals.
Reporting on sex	In this study just female mice were used, due to animal welfare reasons.
Field-collected samples	The study did not involve samples from the field.
Ethics oversight	All experimental procedures including animals were performed in accordance with the protocols approved by the Cantonal Veterinary Office Zurich (license number ZH105/2021).

Note that full information on the approval of the study protocol must also be provided in the manuscript.

Clinical data

Policy information about clinical studies

All manuscripts should comply with the ICMJEguidelines for publication of clinical research and a completedCONSORT checklist must be included with all submissions.

Clinical trial registration	*Provide the trial registration number from ClinicalTrials.gov or an equivalent agency.*
Study protocol	*Note where the full trial protocol can be accessed OR if not available, explain why.*
Data collection	*Describe the settings and locales of data collection, noting the time periods of recruitment and data collection.*
Outcomes	*Describe how you pre-defined primary and secondary outcome measures and how you assessed these measures.*

Dual use research of concern

Policy information about dual use research of concern

Hazards

Could the accidental, deliberate or reckless misuse of agents or technologies generated in the work, or the application of information presented in the manuscript, pose a threat to:

No	Yes	
x	☐	Public health
x	☐	National security
x	☐	Crops and/or livestock
x	☐	Ecosystems
x	☐	Any other significant area

Experiments of concern

Does the work involve any of these experiments of concern:

No	Yes	
x	☐	Demonstrate how to render a vaccine ineffective
x	☐	Confer resistance to therapeutically useful antibiotics or antiviral agents
x	☐	Enhance the virulence of a pathogen or render a nonpathogen virulent
x	☐	Increase transmissibility of a pathogen
x	☐	Alter the host range of a pathogen
x	☐	Enable evasion of diagnostic/detection modalities
x	☐	Enable the weaponization of a biological agent or toxin
x	☐	Any other potentially harmful combination of experiments and agents

Plants

Seed stocks	*Report on the source of all seed stocks or other plant material used. If applicable, state the seed stock centre and catalogue number. If plant specimens were collected from the field, describe the collection location, date and sampling procedures.*
Novel plant genotypes	*Describe the methods by which all novel plant genotypes were produced. This includes those generated by transgenic approaches, gene editing, chemical/radiation-based mutagenesis and hybridization. For transgenic lines, describe the transformation method, the number of independent lines analyzed and the generation upon which experiments were performed. For gene-edited lines, describe the editor used, the endogenous sequence targeted for editing, the targeting guide RNA sequence (if applicable) and how the editor was applied.*
Authentication	*Describe any authentication procedures for each seed stock used or novel genotype generated. Describe any experiments used to assess the effect of a mutation and, where applicable, how potential secondary effects (e.g. second site T-DNA insertions, mosiacism, off-target gene editing) were examined.*

ChIP-seq

Data deposition

☐ Confirm that both raw and final processed data have been deposited in a public database such as GEO.

☐ Confirm that you have deposited or provided access to graph files (e.g. BED files) for the called peaks.

Data access links<br>*May remain private before publication.*	*For "Initial submission" or "Revised version" documents, provide reviewer access links. For your "Final submission" document, provide a link to the deposited data.*
Files in database submission	*Provide a list of all files available in the database submission.*
Genome browser session<br>(e.g. UCSC)	*Provide a link to an anonymized genome browser session for "Initial submission" and "Revised version" documents only, to enable peer review. Write "no longer applicable" for "Final submission" documents.*

Methodology

Replicates	*Describe the experimental replicates, specifying number, type and replicate agreement.*
Sequencing depth	*Describe the sequencing depth for each experiment, providing the total number of reads, uniquely mapped reads, length of reads and whether they were paired- or single-end.*
Antibodies	*Describe the antibodies used for the ChIP-seq experiments; as applicable, provide supplier name, catalog number, clone name, and lot number.*
Peak calling parameters	*Specify the command line program and parameters used for read mapping and peak calling, including the ChIP, control and index files used.*

Data quality	*Describe the methods used to ensure data quality in full detail, including how many peaks are at FDR 5% and above 5-fold enrichment.*
Software	*Describe the software used to collect and analyze the ChIP-seq data. For custom code that has been deposited into a community repository, provide accession details.*

Flow Cytometry

Plots

Confirm that:

- [x] The axis labels state the marker and fluorochrome used (e.g. CD4-FITC).
- [x] The axis scales are clearly visible. Include numbers along axes only for bottom left plot of group (a 'group' is an analysis of identical markers).
- [ ] All plots are contour plots with outliers or pseudocolor plots.
- [x] A numerical value for number of cells or percentage (with statistics) is provided.

Methodology

Sample preparation	Tail tissue was minced and incubated in collagenase II (5 mg/ml) and DNase I (0.05 mg/ml) (both Sigma-Aldrich) in RPMI medium (Gibco) for 30 minutes under agitation at 37°C. Cell suspensions were filtered through 70-µm and 40-µm cell strainers and resuspended in FACS buffer (2% FBS, 2 mM EDTA in PBS). 106 cells/ml were stimulated for 5 h with 100 ng/mL of PMA (Merck) and 1 µg/mL ionomycin (Merck) in the presence of 1 µL/mL GolgiPlug/GolgiStop (BD Biosciences). Subsequently, cells were washed with PBS, and non-specific antibody binding was blocked using mouse TruStain FcX (Biolegend). Cells were incubated for 25 min at 4°C with antibodies for the surface staining. Prior to intracellular labeling, cells were fixed and permeabilized with fixation/permeabilization solution (Thermo Fisher) and stained over night at 4°C.<br><br>Single-cell suspensions isolated from blood were stained for surface markers followed by intracellular staining for Foxp3 using a Foxp3/Transcription Factor Staining Set (eBioscience) according to the manufacturer’s guidelines.<br><br>Blood was diluted with an equal volume of PBS and subsequently was layered carefully over Ficoll in a centrifuge tube without intermixing, followed by centrifugation at 400g for 30 min at 20 °C. PBMCs and platelets, were collected from the interface between plasma and Ficoll layers. To remove platelets, PBMCs were centrifuged in PBS at 300g for 10 min at 20 °C.
Instrument	For the acquisition of human and murine PBMCS a Cytoflex S (Beckman Coulter) apparatus with Cytexpert software (version 2.1.3.22) was used. Single cell suspension from lymphedematous tiussue wereacquired with a Cytek Aurora flow cytometer with SpectroFlo® software (Cytek Biosciences).
Software	Flow cytometry data were analyzed with FlowJo 10.8.1. and statistical analysis was done with GraphPad Prism (Version 9.0)
Cell population abundance	The ceell population abundance is clearly shown in the main figures (cells per all single cells) and in suppl. information (absolute cell counts per g tissue or ml blood).
Gating strategy	*Describe the gating strategy used for all relevant experiments, specifying the preliminary FSC/SSC gates of the starting cell population, indicating where boundaries between "positive" and "negative" staining cell populations are defined.*

- [x] Tick this box to confirm that a figure exemplifying the gating strategy is provided in the Supplementary Information.

Magnetic resonance imaging

Experimental design

Design type	*Indicate task or resting state; event-related or block design.*
Design specifications	*Specify the number of blocks, trials or experimental units per session and/or subject, and specify the length of each trial or block (if trials are blocked) and interval between trials.*
Behavioral performance measures	*State number and/or type of variables recorded (e.g. correct button press, response time) and what statistics were used to establish that the subjects were performing the task as expected (e.g. mean, range, and/or standard deviation across subjects).*

Acquisition

Imaging type(s) | *Specify: functional, structural, diffusion, perfusion.*

Field strength | *Specify in Tesla*

Sequence & imaging parameters | *Specify the pulse sequence type (gradient echo, spin echo, etc.), imaging type (EPI, spiral, etc.), field of view, matrix size, slice thickness, orientation and TE/TR/flip angle.*

Area of acquisition | *State whether a whole brain scan was used OR define the area of acquisition, describing how the region was determined.*

Diffusion MRI ☐ Used ☐ Not used

Preprocessing

Preprocessing software | *Provide detail on software version and revision number and on specific parameters (model/functions, brain extraction, segmentation, smoothing kernel size, etc.).*

Normalization | *If data were normalized/standardized, describe the approach(es): specify linear or non-linear and define image types used for transformation OR indicate that data were not normalized and explain rationale for lack of normalization.*

Normalization template | *Describe the template used for normalization/transformation, specifying subject space or group standardized space (e.g. original Talairach, MNI305, ICBM152) OR indicate that the data were not normalized.*

Noise and artifact removal | *Describe your procedure(s) for artifact and structured noise removal, specifying motion parameters, tissue signals and physiological signals (heart rate, respiration).*

Volume censoring | *Define your software and/or method and criteria for volume censoring, and state the extent of such censoring.*

Statistical modeling & inference

Model type and settings | *Specify type (mass univariate, multivariate, RSA, predictive, etc.) and describe essential details of the model at the first and second levels (e.g. fixed, random or mixed effects; drift or auto-correlation).*

Effect(s) tested | *Define precise effect in terms of the task or stimulus conditions instead of psychological concepts and indicate whether ANOVA or factorial designs were used.*

Specify type of analysis: ☐ Whole brain ☐ ROI-based ☐ Both

Statistic type for inference (See Eklund et al. 2016) | *Specify voxel-wise or cluster-wise and report all relevant parameters for cluster-wise methods.*

Correction | *Describe the type of correction and how it is obtained for multiple comparisons (e.g. FWE, FDR, permutation or Monte Carlo).*

Models & analysis

n/a	Involved in the study
☐	☐ Functional and/or effective connectivity
☐	☐ Graph analysis
☐	☐ Multivariate modeling or predictive analysis

Functional and/or effective connectivity | *Report the measures of dependence used and the model details (e.g. Pearson correlation, partial correlation, mutual information).*

Graph analysis | *Report the dependent variable and connectivity measure, specifying weighted graph or binarized graph, subject- or group-level, and the global and/or node summaries used (e.g. clustering coefficient, efficiency, etc.).*

Multivariate modeling and predictive analysis | *Specify independent variables, features extraction and dimension reduction, model, training and evaluation metrics.*
